# Probe dependence of allosteric enhancers on the binding affinity of adenosine A_1_‐receptor agonists at rat and human A_1_‐receptors measured using NanoBRET

**DOI:** 10.1111/bph.14575

**Published:** 2019-03-06

**Authors:** Samantha L. Cooper, Mark Soave, Manuela Jörg, Peter J. Scammells, Jeanette Woolard, Stephen J. Hill

**Affiliations:** ^1^ Division of Physiology, Pharmacology and Neuroscience, School of Life Sciences University of Nottingham Nottingham UK; ^2^ Centre of Membrane Proteins and Receptors University of Birmingham and University of Nottingham The Midlands UK; ^3^ Medicinal Chemistry, Monash Institute of Pharmaceutical Sciences Monash University Parkville Victoria Australia

## Abstract

**Background and Purpose:**

Adenosine is a local mediator that regulates a number of physiological and pathological processes via activation of adenosine A_1_‐receptors. The activity of adenosine can be regulated at the level of its target receptor via drugs that bind to an allosteric site on the A_1_‐receptor. Here, we have investigated the species and probe dependence of two allosteric modulators on the binding characteristics of fluorescent and nonfluorescent A_1_‐receptor agonists.

**Experimental Approach:**

A Nano‐luciferase (Nluc) BRET (NanoBRET) methodology was used. This used N‐terminal Nluc‐tagged A_1_‐receptors expressed in HEK293T cells in conjunction with both fluorescent A_1_‐receptor agonists (adenosine and NECA analogues) and a fluorescent antagonist CA200645.

**Key Results:**

PD 81,723 and VCP171 elicited positive allosteric effects on the binding affinity of orthosteric agonists at both the rat and human A_1_‐receptors that showed clear probe dependence. Thus, the allosteric effect on the highly selective partial agonist capadenoson was much less marked than for the full agonists NECA, adenosine, and CCPA in both species. VCP171 and, to a lesser extent, PD 81,723, also increased the specific binding of three fluorescent A_1_‐receptor agonists in a species‐dependent manner that involved increases in *B*_max_ and p*K*_D_.

**Conclusions and Implications:**

These results demonstrate the power of the NanoBRET ligand‐binding approach to study the effect of allosteric ligands on the binding of fluorescent agonists to the adenosine A_1_‐receptor in intact living cells. Furthermore, our studies suggest that VCP171 and PD 81,723 may switch a proportion of A_1_‐receptors to an active agonist conformation (R*).

**Abbreviations**
AAG‐ABEA: (BY630‐X‐(*D*)‐A‐(*D*)‐A‐G‐ABEA)
ABA: (ABA‐X‐BY630)
ABEA: (ABEA‐X‐BY630)
CA200645: xanthine amine congener‐β‐alanine,β‐alanine‐BODIPY 630/650
Capadenoson: 2‐amino‐6‐[[2‐(4‐chlorophenyl)‐1,3‐thiazol‐4‐yl]methylsulfanyl]‐4‐[4‐(2‐hydroxyethoxy)phenyl]pyridine‐3,5‐dicarbonitrile
CCPA: (2‐chloro‐*N* ^6^‐cyclopentyladenosine)
DPCPX: 1,3‐dipropyl‐8‐cyclopentylxanthine
MRS1220: *N*‐[9‐chloro‐2‐(furan‐2‐yl)‐[1,2,4]triazolo[1,5‐*c*]quinazolin‐5‐yl]‐2‐phenylacetamide
NECA: 5′‐(*N*‐ethylcarboxamido)adenosine
Nluc: Nano‐luciferase
Nluc‐A_1_R: Nanoluc‐labelled A_1_ adenosine receptor
PD 81,723: (2‐amino‐4,5‐dimethylthiophen‐3‐yl)(3‐(trifluoromethyl)phenyl)methanone
VCP171: (2‐amino‐4‐(3‐(trifluoromethyl)phenyl)thiophen‐3‐yl)(phenyl)methanone
VCP746: 4‐(5‐amino‐4‐benzoyl‐3‐(3‐(trifluoromethyl)phenyl)thiophen‐2‐yl)‐*N*‐(6‐(9‐((2*R*,3*R*,4*S*,5*R*)‐3,4‐dihydroxy‐5‐(hydroxylmethyl)tetrahydro‐furan‐2‐yl)‐9*H*‐purin‐6‐ylamino)hexyl)benzamide

**What is already known**

- Adenosine is a local mediator that regulates physiological processes via activation of adenosine A_1_‐receptors.
- Agonist activity can be regulated by drugs that bind to an allosteric site on the A_1_‐receptor.

**What this study adds**

- This study demonstrates the power of fluorescent ligand NanoBRET approaches to study allosterism in cells.
- Positive allosteric modulators can switch a proportion of A_1_‐receptors to an active agonist‐binding conformation.

**What is the clinical significance**

- This study provides insights into allosteric mechanisms that may provide new opportunities for drug discovery.

## 1 INTRODUCTION

Adenosine is a local reactive metabolite that has a major role in regulating a number of physiological and pathological processes including inflammation, hypoxia, and cardiovascular regulation (Fredholm, Ijzerman, Jacobson, Linden, & Müller, 2011). Adenosine acts via four specific GPCRs, which have been denoted adenosine A_1_‐, A_2A_‐, A_2B_‐, and A_3_‐receptors (Fredholm et al., 2011). The A_1_‐ and A_3_‐receptors preferentially couple to G_i/o_ proteins and have an inhibitory action on adenylyl cyclase activity whilst the A_2A_‐ and A_2B_‐receptors couple to G_s_ proteins and stimulate cAMP formation (Fredholm et al., 2011; Müller & Jacobson, 2011). The crystal structures of the A_2A_‐receptor in both antagonist (Jaakola et al., 2008) and agonist (Xu et al., 2011) bound conformations have been determined, and very recently, the structure of the adenosine A_1_‐receptor has also been solved (Cheng et al., 2017; Glukhova et al., 2017), including an adenosine‐bound A_1_‐receptor in complex with a G_i_‐protein (Draper‐Joyce et al., 2018).

Numerous selective agonists and antagonists for each adenosine receptor subtype are now available for the study of receptor function (see Fredholm et al., 2011; Müller & Jacobson, 2011). In the case of the adenosine A_1_‐receptor, a number of compounds have previously undergone evaluation for cardiovascular disease indications such as paroxysmal supraventricular tachycardia, atrial fibrillation, and angina pectoris (Müller & Jacobson, 2011). At the present time, the A_1_‐receptor partial agonist neladenoson is undergoing clinical trial for heart failure (Meibom et al., 2017). However, the ubiquitous distribution of adenosine receptors in the body can often limit therapeutic application because of the effects of adenosine ligands on the same receptor in a different tissue or cell type (Müller & Jacobson, 2011).

Activation of cell surface adenosine receptors by endogenous adenosine requires it to be available at the extracellular surface of cells. Extracellular adenosine can rise as a consequence of several pathways (Fredholm et al., 2011). It can be formed intracellularly following various metabolic processes and be exported from cells via membrane transporters, or it can be formed in the extracellular space from adenine nucleotides released from cells. Once ATP or ADP is released, the nucleotide is broken down by nucleoside triphosphate diphosphohydrolases (e.g., CD39) and then ecto‐5′‐nucleotidase (CD73) to adenosine (Fredholm et al., 2011; Knapp et al., 2012). The intricacies of localized extracellular release of adenine nucleotides and subsequent production of adenosine following CD73 activity has recently provided insights into the role of adenosine A_1_‐receptors in mediating localized analgesia in animals and humans (Goldman et al., 2010; Sowa, Voss, & Zylka, 2010; Street & Zylka, 2011). In addition, there is increasing evidence that adenosine A_1_‐receptors may be involved in promoting angiogenesis and the release of VEGF in response to local hypoxia and neoplasia (Clark et al., 2007; Merighi et al., 2009).

From the foregoing argument, it is clear that localized regulation of adenosine production may have important therapeutic implications. One way in which the activity of endogenous adenosine can be subtly regulated at the level of its target receptor is via drugs that bind to an allosteric site on the receptor. These allosteric modulators act to enhance or inhibit the binding of adenosine to its receptor binding site (the orthosteric site) and/or change the resulting functional response (Hill, May, Kellam, & Woolard, 2014). Some of the earliest allosteric modulators, such as PD 81,723, were discovered for the adenosine A_1_‐receptor (Bruns & Fergus, 1990; Bruns et al., 1990; Göblyös & IJzerman, 2011; Kimatrai‐Salvador, Baraldi, & Romagnoli, 2012). VCP171, [(2‐amino‐4‐(3‐(trifluoromethyl)phenyl)thiophen‐3‐yl)(phenyl)methanone], has also recently been described as a novel 2‐amino‐3‐benzoylthiophene positive allosteric modulator (Aurelio et al., 2009, 2010; Valant et al., 2010; Vincenzi et al., 2014) and has been investigated in a rat model of neuropathic pain (Imlach, Bhola, May, Christopoulos, & Christie, 2015).

The potential for allosteric enhancers to provide highly localized augmentation of adenosine actions on target receptors is well established (Hill et al., 2014). However, the in vivo actions of allosteric regulators have not been extensively investigated, and there is a need to evaluate the potential for these small molecules to augment specific actions of adenosine in particular organs or cell types in a whole animal setting. Some limited success has been achieved in vivo with PD 81,723. Activation of adenosine A_1_‐receptors has been shown to protect against renal ischaemia/reperfusion injury in experimental animals (Lee & Emala, 2000; Lee, Gallos, Nasr, & Emala, 2004; Park et al., 2012). However, despite the high homology between the species homologues of the A_1_‐receptor, there is evidence for species differences in the affinity of certain adenosine receptor ligands (Müller & Jacobson, 2011; Szymańska et al., 2016).

The aim of the present study was to compare the effect of PD 81,723 and VCP171 on the human and rat adenosine A_1_‐receptors expressed in HEK293T cells and to evaluate the extent to which they exhibit probe and species dependence in a whole cell environment. To do this, we made use of the recently established Nano‐luciferase (Nluc) BRET methodology (NanoBRET; Stoddart, Johnstone, et al., 2015; Stoddart, Vernall, Briddon, Kellam, & Hill, 2015) using N‐terminal Nluc‐tagged A_1_‐receptors in conjunction with fluorescent A_1_‐receptor agonists and antagonists (Stoddart, Johnstone, et al., 2015). These fluorescent ligands included the xanthine amine congener based antagonist ligand CA200645 (Corriden, Kilpatrick, Kellam, Briddon, & Hill, 2014; Stoddart, Johnstone, et al., 2015) and fluorescent A_1_‐agonists based on adenosine (ABA‐X‐BY630; Middleton et al., 2007; May, Self, Briddon, & Hill, 2010) or NECA (ABEA‐X‐BY630, Cordeaux, Briddon, Alexander, Kellam, & Hill, 2008; Stoddart, Johnstone, et al., 2015; BY630‐X‐(D)‐A‐(D)‐A‐G‐ABEA; Stoddart, Vernall, et al., 2015).

## 2 METHODS

### 2.1 Constructs, cell lines, and cell culture

Human and rat Nluc‐labelled adenosine A_1_‐receptor (Nluc‐A_1_R) constructs were generated as previously described by Stoddart, Johnstone, et al. (2015). In brief, the full‐length sequence of Nluc luciferase from the pNL1.1 vector (Promega) was amplified and fused in‐frame with the membrane signal sequence of the 5‐HT_3A_ membrane localization signal sequence (pcDNA3.1 sig‐Nluc; Soave, Stoddart, Brown, Woolard, & Hill, 2016). This was fused to the full‐length human or rat sequence of the adenosine A_1_‐receptor (with the methionine start signal removed) to the 3′ end of the sig‐Nluc in pcDNA3.1. The resulting fusion protein contained a Gly‐Ser linker between the Nluc open reading frame and the human or rat A_1_ open reading frame. This resulted in the human and rat Nluc‐A_1_R constructs.

### 2.2 Cultured cells

HEK293T cells (ATCC Cat# CRL‐3216, RRID:CVCL_0063) were maintained in DMEM supplemented with 2 mM L‐glutamine and 10% fetal calf serum at 37°C 5% CO_2_. Once 70–80% confluent, cells were dislodged from the flask surface by gentle shaking after incubation in 0.25% trypsin and collected following centrifugation at 1000× *g* for 5 min. Cells were then seeded at 2–5 × 10,000 cells cm^‐2^. Mixed population human Nluc‐A_1‐_AR and rat Nluc‐A_1‐_AR cell lines were generated using Fugene HD (Promega) according to the manufacturer's instructions, and cells were then subjected to 1 mg/mL G418‐selection pressure for 2 weeks.

### 2.3 BRET human and rat Nluc‐A_1_ R ligand‐binding assays

The fluorescent antagonist saturation, competition‐binding, allosteric modulator binding cooperativity, and the fluorescent agonist saturations in the presence/absence of allosteric modulator assays were performed on the stably transfected HEK293T cells expressing human or rat Nluc‐A_1_R. The cells were seeded 24 hr before experimentation in white walled, poly‐d‐lysine coated 96‐well microplates (Thermo Scientific, Loughborough, UK) at a density of 25,000 cells per well.

The medium was replaced with HEPES‐buffered saline solution (145 nM NaCl, 5 mM KCl, 1.7 mM CaCl_2_, 1 mM MgSO_4_, 10 mM HEPES, 2 mM sodium pyruvate, 1.5 mM NaHCO_3_, 10 mM d‐glucose, pH 7.2–7.45), with the required concentration of fluorescent ligand, competing ligand, and/or allosteric modulator. For each experiment, ligands were added simultaneously, and the 96‐well plate was incubated for 1 hr at 37°C (no CO_2_). Following this, the Nluc substrate furimazine (Promega) was added to give a final concentration of 10 μM and then incubated for 5 min at 37°C. For all experiments, the luminescence and resulting BRET were measured using the PHERAstar FS plate reader (BMG Labtech) using filtered light emissions at 460 nm (80 nm bandpass) and >610 nm (longpass) at room temperature. The raw BRET ratio was calculated by dividing the >610 nm emission by the 460 nm emission.

### 2.4 Data analysis

Data were presented and analysed using Prism 7 software (GraphPad software, San Diego, CA, USA).

Saturation‐binding curves were simultaneously fitted to obtain the total and non‐specific components using the following equation:

$$\text{BRET ratio}\;=\;\frac{B_{\max}\times \;\left[B\right]}{\left[B\right]+K_{D}}+\left(\left(M\times \left[B\right]\right)+C\right),$$

where *B*_max_ is the maximal level of specific binding, [*B*] is the concentration of fluorescent ligand in nM, *K*_D_ is the equilibrium dissociation constant in nM, *M* is the slope of the linear non‐specific binding component, and *C* is the *y*‐axis intercept.

Competition NanoBRET data was fitted using a one‐site sigmoidal competition curve given by the following equation:

$$\%\text{uninhibited binding}=100-\frac{\left(100\times \left[A^{n}\right]\right)}{\left(\left[A^{n}\right]+{\mathrm{IC}}_{50}^{n}\right)}+\mathrm{NS},$$

where [*A*] is the concentration of competing drug, *NS* is the non‐specific binding, *n* is the Hill coefficient, and IC_50_ is the concentration of ligand required to inhibit 50% of the specific binding of the fluorescent ligand.

The IC_50_ values from competition‐binding curves were used to calculate the *K*_i_ of the unlabelled ligands using the Cheng–Prusoff equation:

$$K_{i}=\frac{{\mathrm{IC}}_{50}}{1+\frac{\left[L\right]}{K_{D}}},$$

where [*L*] is the concentration of fluorescent ligand in nM, and *K*_D_ is the dissociation constant of the fluorescent ligand in nM. The *K*_D_ values used were obtained from the saturation‐binding experiments.

Pooled fluorescent agonist saturation assays obtained in the presence and absence of a fixed concentration of allosteric modulator were simultaneously fitted to the following equation:

$$\text{BRET ratio}\;=\;\frac{B_{\max}\times \left[B\right]}{\left[B\right]+K_{D}}+\left(\left(M\times \left[B\right]\right)+C\right).$$

The slope of the non‐specific binding component *M* was kept constant (equivalent to the slope of the binding curve obtained in the presence of 1 μM DPCPX in the same experiments), and a partial *F* test was used to determine whether a significantly better fit was obtained with individual parameters for *B*_max_ and *K*_D_ for each curve (control vs. that obtained in the presence of VCP171 or PD 81,723) when compared with sharing the parameters between curves.

### 2.5 Statistical analysis

The statistical analyses in this study comply with the recommendations on experimental design and analysis in pharmacology (Curtis et al., 2018). Statistical significance was determined by one‐way ANOVA followed by Tukey's post hoc test, partial *F* test, or unpaired Student's *t* test. In all cases, differences were considered significant at *P* < 0.05. All statistical analysis was performed using GraphPad Prism 7.03 (RRID:SCR_002798). In all cases, individual experiments were performed in triplicate, and statistical analysis was performed on the data obtained from five or six repeat experiments.

### 2.6 Materials

Adenosine receptor ligands: adenosine (Cat# A9251), 5′‐*N*‐ethylcarboxamidoadenosine (NECA; Cat# E2387), and (2‐amino‐4,5‐dimethylthiophen‐3‐yl)(3‐(trifluoromethyl)phenyl)methanone (PD 81,723; Cat#P1123) were purchased from Sigma‐Aldrich (Gillingham, UK). 1,3‐Dipropyl‐8‐cyclopentylxanthine (DPCPX; C101), 2‐chloro‐*N* ^6^‐cyclopentxyladenosine (CCPA; Cat# C7938), and *N*‐[9‐chloro‐2‐(furan‐2‐yl)‐[1,2,4]triazolo[1,5‐*c*]quinazolin‐5‐yl]‐2‐phenylacetamide (MRS1220; Cat# M228) were purchased from Tocris Bioscience (Bristol, UK).

(2‐Amino‐4‐(3‐(trifluoromethyl)phenyl)thiophen‐3‐yl)(phenyl)methanone (VCP171) and 4‐(5‐amino‐4‐benzoyl‐3‐(3‐(trifluoromethyl)phenyl)thiophen‐2‐yl)‐*N*‐(8‐(9‐((2*R*,3*R*,4*S*,5*R*)‐3,4‐dihydroxy‐5‐(hydroxylmethyl)tetrahydro‐furan‐2‐yl)‐9*H*‐purin‐6‐ylamino)hexyl)benzamide (VCP746) were synthesized as previously described by Aurelio et al. (2009) and Valant et al. (2014) respectively. 2‐Amino‐6‐[[2‐(4‐chlorophenyl)‐1,3‐thiazol‐4‐yl]methylsulfanyl]‐4‐[4‐(2‐hydroxyethoxy)phenyl]pyridine‐3,5‐dicarbonitrile (capadenoson) was purchased from Haoyuan Chemexpress (Cat# HY‐14917; Shanghai, China).

The fluorescent A_1‐_receptor antagonist, CA200645, was purchased from Hello Bio (Cat# HB7812; Bristol, UK). The fluorescent A_1‐_receptor agonist, ABA‐X‐BY630 (Briddon et al., 2004), was purchased from CellAura Technologies Ltd. (Nottingham, UK). The fluorescent A_1‐_receptor agonist, ABEA‐X‐BY630, was synthesized as previously described by Middleton et al. (2007). The fluorescent A_1‐_receptor agonist, BY630‐X‐(*D*)‐A‐(*D*)‐A‐G‐ABEA, was synthesized as described by Stoddart, Vernall, et al. (2015). Fugene HD transfection reagent and furimazine were from Promega (Southampton, UK). All other reagents were from Sigma‐Aldrich (Gillingham, UK).

### 2.7 Nomenclature of targets and ligands

Key protein targets and ligands in this article are hyperlinked to corresponding entries in http://www.guidetopharmacology.org/, the common portal for data from the IUPHAR/BPS Guide to PHARMACOLOGY (Harding et al., 2018), and are permanently archived in the Concise Guide to PHARMACOLOGY 2017/18 (Alexander et al., 2017).

## 3 RESULTS

### 3.1 Measurement of the specific binding of CA200645 to rat and human adenosine A_1_‐receptors using NanoBRET

We have recently described a bioluminescence energy transfer approach (NanoBRET) to monitor ligand–receptor interactions in living HEK293T cells expressing the human A_1_‐receptor tagged on its N‐terminus with the luminescence protein Nluc (Stoddart, Johnstone, et al., 2015). Here, we have compared the ligand‐binding characteristics of Nluc‐tagged human and rat adenosine A_1_‐receptors using the fluorescent antagonist ligand CA200645 (Stoddart, Johnstone, et al., 2015). Binding experiments were performed over a large range of concentrations of CA200645 (1–500 nM) and yielded clear saturable components of specific binding for both species receptor homologues with negligible non‐specific binding detected in the presence of a high concentration of the A_1_‐receptor selective antagonist DPCPX (1 μM). The *K*_D_ values obtained for CA200645 for the specific binding component were 33.84 ± 10.15 nM (*n* = 6) and 35.44 ± 4.66 nM (*n* = 6) for the human and rat Nluc‐A_1_‐receptors, respectively.

### 3.2 Inhibition of binding by A_1_‐receptor ligands

**Figure 1:**
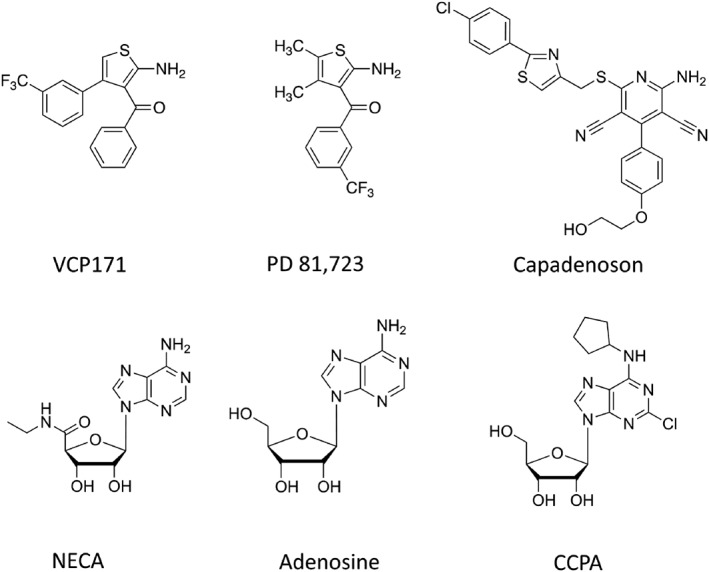
Chemical structures of VCP171, PD 81,723, and A_1_‐receptor agonists

The binding affinities of non‐fluorescent A_1_‐receptor ligands at the two species homologues were then determined from competition‐binding studies in the presence of 25 nM CA200645 (Table 1). Capadenoson, CCPA, NECA, and adenosine (Figure 1) showed similar affinities between the two species (Table 1), with capadenoson being the highest affinity agonist in both species. The selective A_1_‐receptor antagonist DPCPX was a potent inhibitor of CA200645 binding and exhibited a higher affinity for the rat A_1_‐receptor (Table 1). In contrast, the antagonist MRS1220, the allosteric ligand VCP171 and VCP746 (a hybrid molecule made up of adenosine and VCP171; Valant et al., 2014) had slightly higher affinities for the human A_1_‐receptor (Table 1). The other allosteric ligand studied, PD 81,723, was generally a weak direct inhibitor of the binding of CA200645 in both species (Table 1).

**Table 1:** Binding affinities of nine competing ligands determined from inhibition of the specific binding of CA200645 at the human Nluc‐A_1_AR and rat Nluc‐A_1_AR

	Nluc‐human A_1_AR			Nluc‐rat A_1_AR		
	pIC_50_ ± SEM	p*K*_i_ ± SEM	*N*	pIC_50_ ± SEM	p*K*_i_ ± SEM	*N*
Adenosine	4.17 ± 0.08	4.41 ± 0.08	6	4.27 ± 0.08	4.53 ± 0.08	5
Capadenoson	6.61 ± 0.12	6.85 ± 0.12	6	6.73 ± 0.11	6.99 ± 0.11	5
NECA	5.26 ± 0.17	5.50 ± 0.17	6	5.12 ± 0.04	5.39 ± 0.04	5
CCPA	6.15 ± 0.05	6.40 ± 0.05^a^	12	6.32 ± 0.04	6.58 ± 0.04	10
PD 81,723	3.99 ± 0.24	4.23 ± 0.24	6	<4	n.d.	5
VCP171	4.39 ± 0.09	4.63 ± 0.09^a^	6	3.89 ± 0.14	4.15 ± 0.14	5
VCP746	5.25 ± 0.09	5.49 ± 0.09^a^	6	4.76 ± 0.05	5.02 ± 0.06	5
DPCPX	7.89 ± 0.0.7	8.13 ± 0.07^a^	6	8.38 ± 0.15	8.64 ± 0.15	5
MRS1220	6.42 ± 0.07	6.66 ± 0.07^a^	6	5.71 ± 0.24	5.97 ± 0.24	5

*Note*. Data are expressed as mean ± SEM in *n* separate experiments, performed in triplicate. p*K*_i_ values were determined from IC_50_ values using the Cheng–Prusoff equation.

a p*K*_i_ values obtained of competing ligand significantly differ between human Nluc‐A_1_R and rat Nluc‐A_1_R (*P < 0.05; unpaired *t* test).

### 3.3 Allosteric regulation of the inhibition of fluorescent A_1_‐receptor antagonist binding by A_1_‐receptor agonists

**Figure 2:**
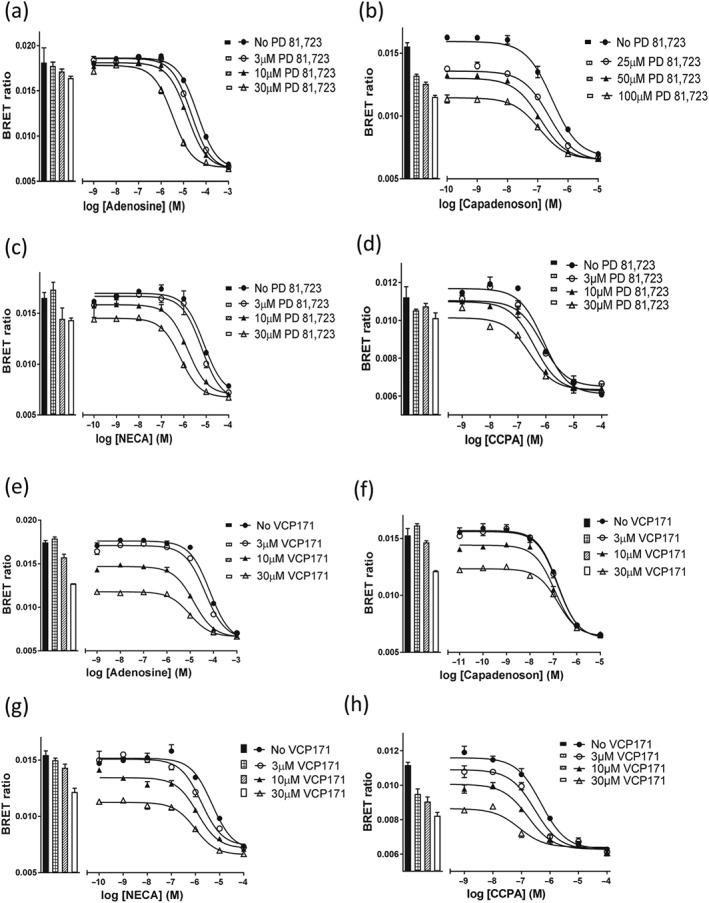
Effect of PD 81,723 and VCP171 on agonist binding to the human Nluc‐A_1_R. The effect of the allosteric modulators PD 81,723 and VCP171 on the ability of adenosine A_1_‐receptor agonists (adenosine, capadenoson, CCPA, and NECA) to inhibit CA200645 (25 nM) binding was monitored using BRET. (a) Adenosine and PD 81,723; (b) capadenoson and PD 81,723; (c) NECA and PD 81,723; (d) CCPA and PD 81,723; (e) adenosine and VCP171; (f) capadenoson and VCP171; (g) NECA and VCP171; and (h) CCPA and VCP171. Figures shown are single representative experiments from five (a, c, g) or six (b, d–f, h) separate experiments that were each performed in triplicate. Data are expressed as mean ± SEM of the triplicate data

To investigate the potential for PD 81,723 and VCP171 (Figure 1) to regulate A_1_‐receptor agonist binding to the human and rat A_1_‐receptors in living cells, we evaluated the effect co‐incubation with increasing concentrations of VCP171 or PD 81,723 on the ability of adenosine, NECA, CCPA, and capadenoson to inhibit the specific binding of CA200645 to Nluc‐tagged A_1_‐receptors. PD 81,723 used at concentrations of 3, 10, or 30 μM shifted the agonist competition curves to the left and produced a decrease in the IC_50_ values for adenosine, CCPA, and NECA binding to the human A_1_‐receptor (Figure 2a,c,d; Table 2), without markedly changing the direct binding of CA200645 alone (Figure 2a,c,d). Significant effects on IC_50_ values were observed with 10 μM PD 81,723 for NECA and 30 μM PD 81,723 for adenosine and CCPA (Table 2). A smaller effect was observed on the A_1_‐receptor selective agonist capadenoson (Albrecht‐Kupper, Leineweber, & Nell, 2012; Tendera et al., 2012), and higher concentrations of PD 81,723 (that also had a direct inhibitory effect on the binding of CA200656 alone) were required to produce a significant change (Figure 2b; Table 2).

**Table 2:** The effect of three different concentrations of allosteric modulator (PD 81,723 or VCP171) on agonist (adenosine, capadenoson, NECA, and CCPA) pIC_50_ values, determined by the inhibition of CA200645 specific binding of at the human or rat Nluc‐A_1_‐AR

	Species	PD 81,723				VCP171			
		0	3 μM	10 μM	30 μM	0	3 μM	10 μM	30 μM
Adenosine	Human	4.18 ± 0.11	4.36 ± 0.14	4.66 ± 0.12	5.07 ± 0.18*	4.23 ± 0.06	4.38 ± 0.06	4.77 ± 0.09*	5.05 ± 0.13*
NECA	Human	5.21 ± 0.08	5.21 ± 0.10	5.72 ± 0.08*	5.99 ± 0.09*	5.37 ± 0.06	5.68 ± 0.05*	5.96 ± 0.04*	6.00 ± 0.05*
CCPA	Human	6.15 ± 0.09	6.29 ± 0.10	6.49 ± 0.12	6.75 ± 0.08*	6.14 ± 0.10	6.47 ± 0.06*	6.68 ± 0.07*	6.80 ± 0.09*
Capadenoson	Human	6.55 ± 0.10	6.67 ± 0.07^a^	6.84 ± 0.08^b^	6.94 ± 0.06^c^, *	6.63 ± 0.09	6.66 ± 0.09	6.71 ± 0.08	6.63 ± 0.07
Adenosine	Rat	4.61 ± 0.37	4.74 ± 0.22	4.85 ± 0.27	5.12 ± 0.35	4.13 ± 0.08	4.66 ± 0.28	5.14 ± 0.15*	4.99 ± 0.28
NECA	Rat	5.43 ± 0.19	5.50 ± 0.23	5.70 ± 0.35	6.00 ± 0.31	5.03 ± 0.10	5.62 ± 0.16	5.95 ± 0.20*	6.13 ± 0.23*
CCPA	Rat	6.28 ± 0.05	6.43 ± 0.03*	6.70 ± 0.02*	6.91 ± 0.04*	6.35 ± 0.07	6.78 ± 0.03*	7.06 ± 0.03*	6.96 ± 0.10*
Capadenoson	Rat	6.56 ± 0.16	6.54 ± 0.12	6.74 ± 0.17	6.71 ± 0.11	6.60 ± 0.07	6.76 ± 0.14	6.79 ± 0.15	6.84 ± 0.16

*Note*. Data are expressed as mean ± SEM in separate experiments (*n* = 5 or 6), performed in triplicate.

a 25 μM PD 81,723.

b 50 μM PD 81,723.

c 100 μM PD 81,723.

* *P* < 0.05, compared to 0 allosteric modulator; one‐way ANOVA, post hoc Tukey's test.

In the case of VCP171, 3, 10, or 30 μM concentrations of this allosteric regulator not only produced a clearer decrease in the specific binding of CA200645 alone to the human A_1_‐receptor but also produced significant decreases in the IC_50_ for adenosine, CCPA, and NECA (Figure 2e,g,h; Table 2) without producing a significant change in the IC_50_ of capadenoson (Figure 2f; Table 2).

**Figure 3:**
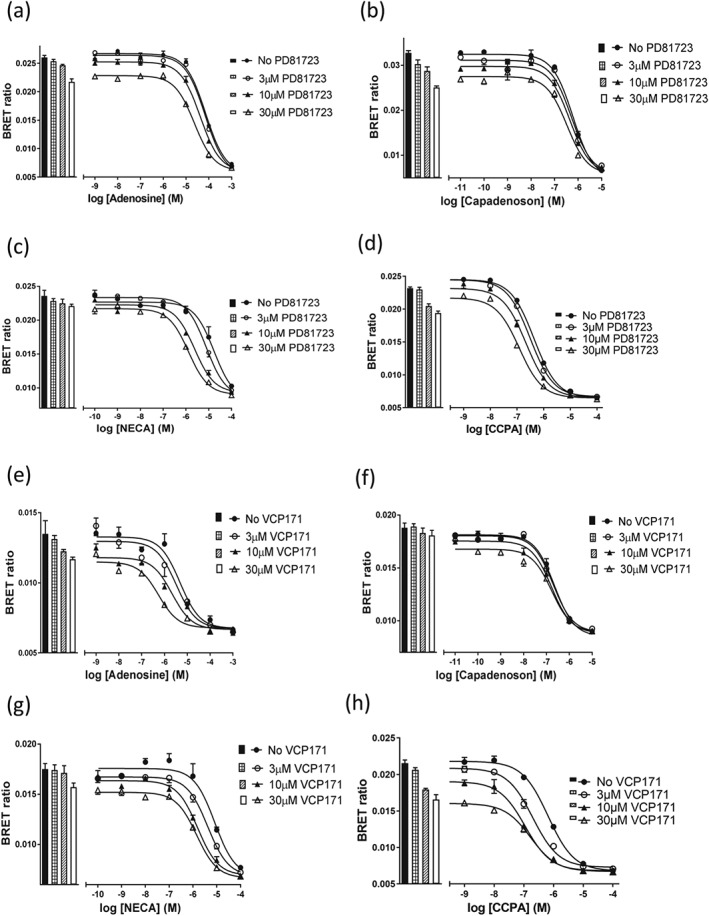
Effect of PD 81,723 and VCP171 on agonist binding to the rat Nluc‐A_1_R. The effect of the allosteric modulators PD 81,723 and VCP171 on the ability of adenosine A_1_‐receptor agonists (adenosine, capadenoson, CCPA, and NECA) to inhibit CA200645 (25 nM) binding was monitored using BRET. (a) Adenosine and PD 81,723; (b) capadenoson and PD 81,723; (c) NECA and PD 81,723; (d) CCPA and PD81,723; (e) adenosine and VCP171; (f) capadenoson and VCP171; (g) NECA and VCP171; and (h) CCPA and VCP171. Figures shown are single representative experiments from five (a–f, h) or six (g) separate experiments that were each performed in triplicate. Data are expressed as mean ± SEM of the triplicate data

At the rat A_1_‐receptor, PD 81,723 (3, 10, or 30 μM) did not significantly alter the IC_50_ values for inhibition of specific CA200645 binding of adenosine, NECA, or capadenoson (Figure 3a,b,c; Table 2), although there was a tendency to produce a small decrease in IC_50_ for adenosine and NECA (Table 2). There was, however, a significant effect on the IC_50_ value of CCPA at 10 and 30 μM PD 81,723 (Table 2; Figure 3d). In contrast, VCP171 (10 or 30 μM) significantly decreased the IC_50_ values of CCPA, NECA, and to a lesser extent adenosine (Figure 3e,g,h) but had no significant effect on the agonist response to capadenoson (Figure 3f; Table 2).

### 3.4 Allosteric effect on the specific binding of fluorescent A_1_‐receptor agonists

**Figure 4:**
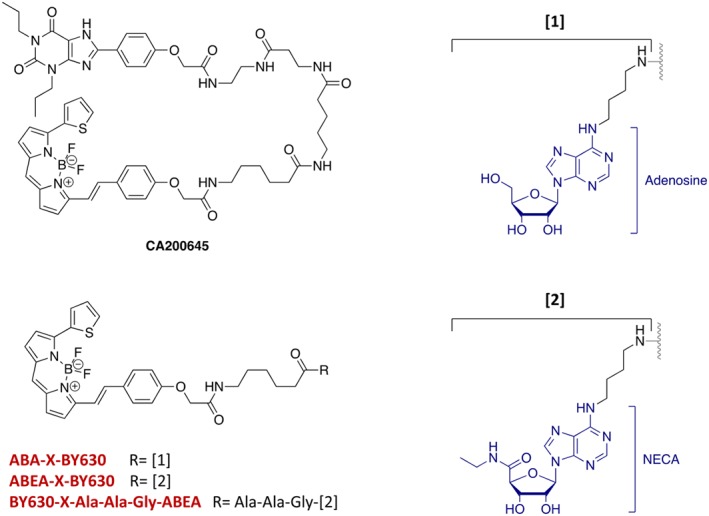
Chemical structure of three fluorescent agonists (BY630‐X‐ABA, BY630‐X‐ABEA, and BY630‐Ala‐Ala‐Gly‐ABEA) and the fluorescent antagonist CA200645

To establish whether a direct action of allosteric regulators could be demonstrated on the binding of fluorescent agonists to the A_1_‐receptor in living cells, we investigated their effect on the binding of the adenosine based fluorescent ligand ABA‐X‐BY630 and two NECA‐based fluorescent derivatives: ABEA‐X‐BY630 and its tripeptide linker variant BY630‐X‐Ala‐Ala‐Gly‐ABEA (AAG‐ABEA‐X‐BY630; Figure 4). ABA‐X‐BY630 is an effective agonist at A_1_‐receptors in mediating inhibition of CRE‐mediated gene expression, calcium mobilization, and ERK1/2 phosphorylation (Briddon et al., 2004; May et al., 2010). Similarly, ABEA‐X‐BY630 and AAG‐ABEA‐X‐BY630 have been shown to be full agonists of A_1_‐receptor‐mediated inhibition of forskolin‐stimulated cAMP accumulation or CRE‐mediated gene expression respectively (Middleton et al., 2007; Stoddart, Vernall, et al., 2015).

**Figure 5:**
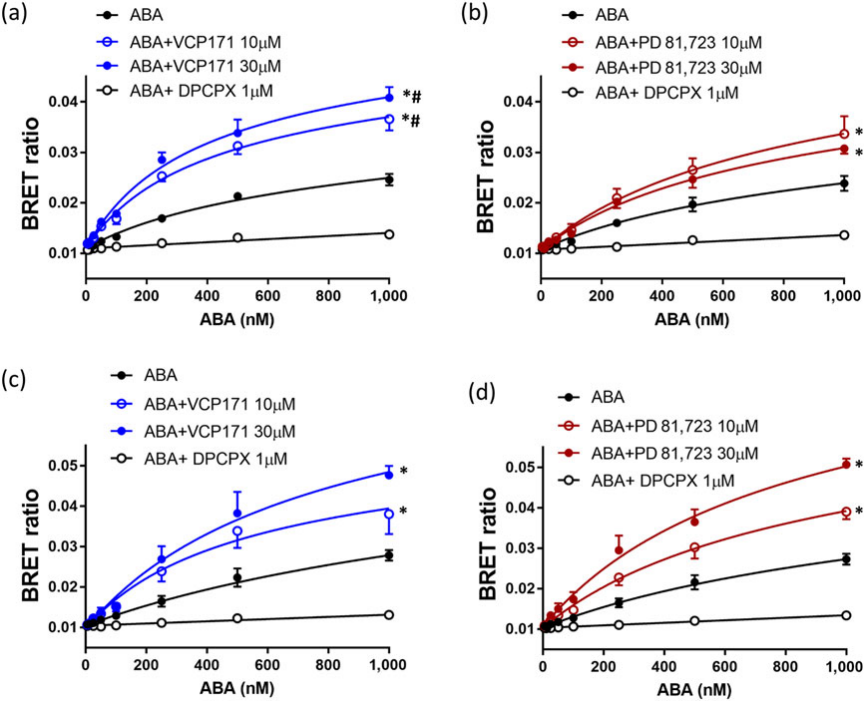
Effect of VCP171 and PD 81,723 on the binding of ABA‐X‐BY630 (ABA) to the human and rat adenosine A_1_‐receptors. Effect of VCP171 (a, c) or PD 81,723 (b, d) on the binding of ABA‐X‐BY630 to the human (a, b) or rat (c, d) A_1_‐receptors. Figures show combined data from five separate experiments (each performed in triplicate). Data are expressed as mean ± SEM. *P < 0.05 fitted parameters (both *K*_D_ and *B*_max_) curves significantly different from control (without allosteric modulator; partial *F* test). ^#^ *P* < 0.05 fitted parameter for *B*_max_ significantly different from control (partial *F* test)

VCP171 produced a significant increase in the specific binding of the adenosine analogue ABA‐X‐BY630 to both the human (Figure 5a) and rat A_1_‐receptors (Figure 5c). Partial *F* test analysis of the non‐linear regression fits to the combined data shown in Figure 3 confirmed that the two binding parameters (p*K*_D_ and *B*_max_) differed significantly between the control and VCP171 (10 or 30 μM) curves (i.e., they could not be shared; Figure 5a,c). In the case of the human receptor, this could also be ascribed to a significant change in the *B*_max_ value (partial *F* test). Analysis of the mean parameters from the individual repeat experiments (Tables 3 and 4) confirmed a significant increase in *B*_max_ and p*K*_D_ values for the human (Table 3) but not the rat A_1_‐receptor (Table 4). Smaller but still significant increases (partial *F* test) in specific binding of ABA‐X‐BY630 were also observed with PD 81,723 in the human (Figure 5b) and rat (Figure 5d). However, neither effect could be reliably ascribed to a change in an individual binding parameter (*B*_max_ or p*K*_D_). From the analysis of individual experiments, a significant change was only observed in p*K*_D_ of the rat A_1_‐receptor at 10 μM PD 81,723 (Table 4).

**Table 3:** The effect of three different concentrations of allosteric modulator (PD 81,723 or VCP171) on the binding of the fluorescent agonists ABA‐X‐BY630, ABEA‐X‐BY630, and BY630‐X‐AAG‐ABEA to the human Nluc‐A_1_AR

Human Nluc A_1_R		VCP171				PD 81,723			
Fluorescent ligand	p*K*_D_ or *B*_max_	0	10 μM	30 μM	*N*	0	10 μM	30 μM	*N*
ABA‐X‐BY630	p*K*_D_	6.23 ± 0.05	6.44 ± 0.08*	6.46 ± 0.05*	5	6.11 ± 0.02	6.15 ± 0.09	6.11 ± 0.20	5
ABA‐X‐BY630	*B*_max_	0.019 ± 0.002	0.033 ± 0.004*	0.036 ± 0.003*	5	0.019 ± 0.002	0.035 ± 0.005	0.035 ± 0.008	5
ABEA‐X‐BY630	p*K*_D_	5.99 ± 0.15	6.43 ± 0.05*	6.43 ± 0.08*	6	6.01 ± 0.15	5.96 ± 0.11	5.94 ± 0.19	5
ABEA‐X‐BY630	*B*_max_	0.006 ± 0.001	0.013 ± 0.002*	0.016 ± 0.002*	6	0.007 ± 0.001	0.012 ± 0.003	0.016 ± 0.004	5
BY630‐X‐AAG‐ABEA	p*K*_D_	6.17 ± 0.16	6.97 ± 0.16*	6.97 ± 0.15*	6	6.11 ± 0.20	6.35 ± 0.17	6.49 ± 0.16	5
BY630‐X‐AAG‐ABEA	*B*_max_	0.019 ± 0.001	0.021 ± 0.001	0.024 ± 0.001*	6	0.021 ± 0.003	0.022 ± 0.002	0.24 ± 0.002	5

*Note*. p*K*_D_ and *B*_max_ values were obtained from individual experiments. Data are expressed as mean ± SEM obtained in *N* separate experiments. Each individual experiment was performed in triplicate.

* *P* < 0.05, compared to 0 allosteric modulator; one‐way ANOVA, post hoc Tukey's test.

**Table 4:** The effect of three different concentrations of allosteric modulator (PD 81,723 or VCP171) on the binding of the fluorescent agonists ABA‐X‐BY630, ABEA‐X‐BY630, and BY630‐X‐AAG‐ABEA to the rat Nluc‐A_1_AR

Rat Nluc A_1_R		VCP171				PD 81,723			
Fluorescent ligand	p*K*_D_ or *B*_max_	0	10 μM	30 μM	*N*	0	10 μM	30 μM	*N*
ABA‐X‐BY630	p*K*_D_	6.05 ± 0.30	6.31 ± 0.18	6.16 ± 0.19	5	6.02 ± 0.14	6.25 ± 0.07	6.33 ± 0.11	5
ABA‐X‐BY630	*B*_max_	0.031 ± 0.014	0.048 ± 0.009	0.072 ± 0.021	5	0.031 ± 0.002	0.040 ± 0.005	0.054 ± 0.007*	5
ABEA‐X‐BY630	p*K*_D_	6.05 ± 0.19	6.18 ± 0.08	6.22 ± 0.07	5	5.74 ± 0.14	5.86 ± 0.13	5.83 ± 0.22	6
ABEA‐X‐BY630	*B*_max_	0.010 ± 0.001	0.021 ± 0.002*	0.026 ± 0.003*	5	0.015 ± 0.002	0.018 ± 0.002	0.032 ± 0.01	6
BY630‐X‐AAG‐ABEA	p*K*_D_	5.75 ± 0.17	6.60 ± 0.12*	6.49 ± 0.15*	5	5.79 ± 0.07	5.74 ± 0.15	6.01 ± 0.07	5
BY630‐X‐AAG‐ABEA	*B*_max_	0.026 ± 0.003	0.023 ± 0.003	0.034 ± 0.005	5	0.016 ± 0.002	0.038 ± 0.008	0.036 ± 0.007	5

*Note*. p*K*_D_ and *B*_max_ values were obtained from individual experiments. Data are expressed as mean ± SEM obtained in *N* separate experiments. Each individual experiment was performed in triplicate.

* *P* < 0.05 compared to 0 allosteric modulator; one‐way ANOVA, post hoc Tukey's test.

**Figure 6:**
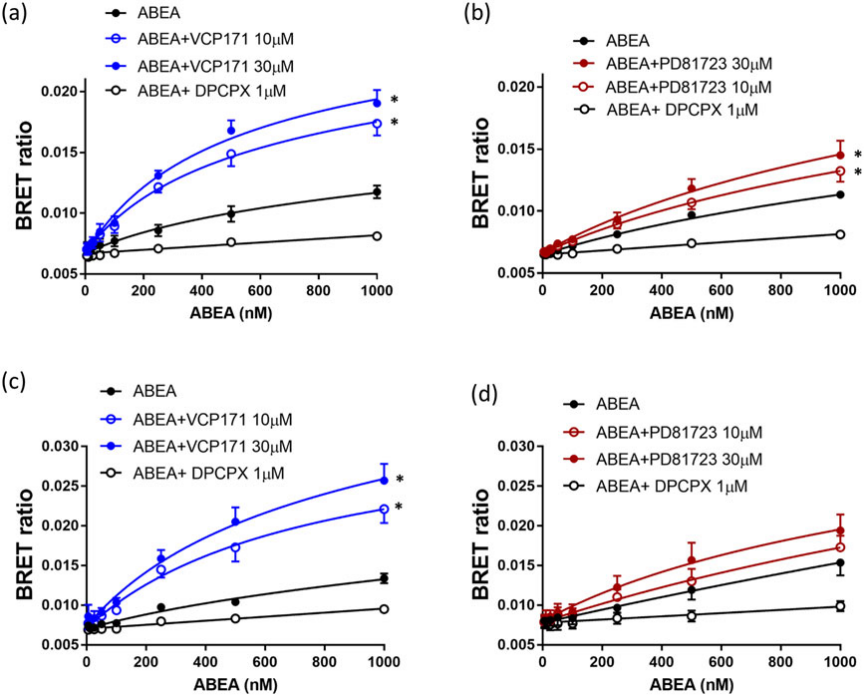
Effect of VCP171 and PD 81,723 on the binding of ABEA‐X‐BY630 (ABEA) to the human and rat adenosine A_1_‐receptors. Effect of VCP171 (a, c) or PD 81,723 (b, d) on the binding of ABEA‐X‐BY630 to the human (a, b) or rat (c, d) A_1_‐receptors. Figures show combined data from six (a, d) or five (b, c) separate experiments (each performed in triplicate). Data are expressed as mean ± SEM. *P < 0.05 fitted parameters (both *K*_D_ and *B*_max_) curves significantly different from control (without allosteric modulator; partial *F* test)

In the case of the NECA derivative ABEA‐X‐BY630, significant increases in specific binding were detected with VCP171 at both A_1_‐receptor species homologues (Figure 6a,c). At the human A_1_‐receptor, this was due to significant changes in both p*K*_D_ and *B*_max_ (Table 3), whereas for the rat homologue, it was more dependent upon an increase in *B*_max_ (Table 4). PD 81,723 did not significantly change any of the binding parameters for the human (Table 3) or rat (Table 4) A_1_‐receptors, but there was a very small elevation in overall specific binding at the human A_1_‐receptor but not the rat (Figure 6b,d).

**Figure 7:**
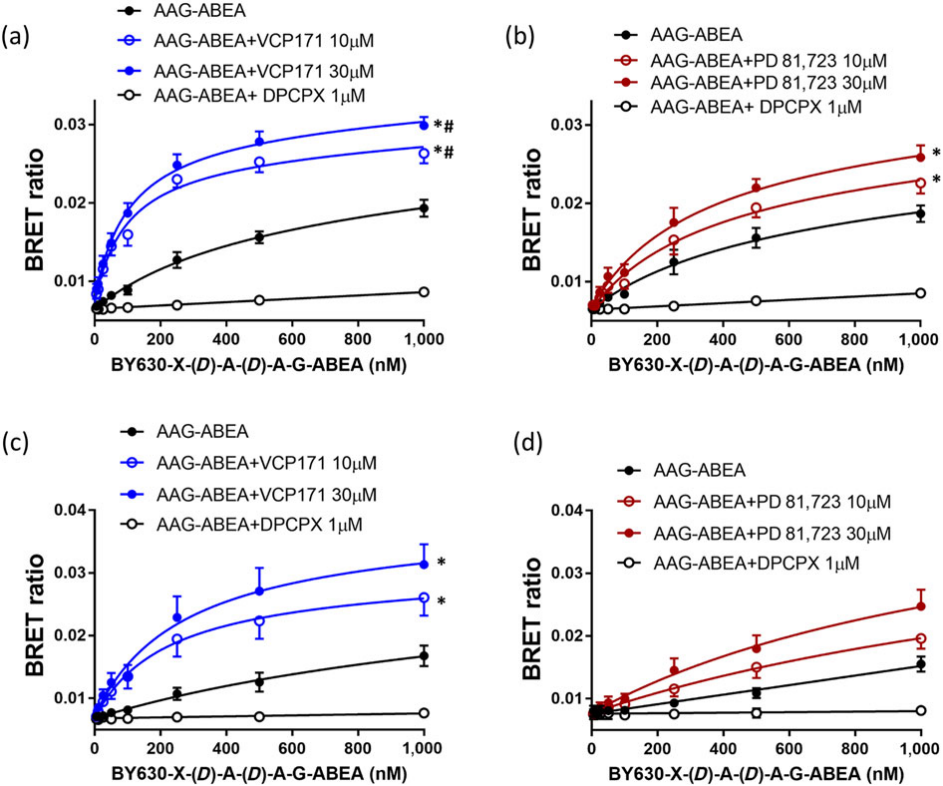
Effect of VCP171 and PD 81,723 on the binding of BY630‐X‐(*D*)‐A‐(*D*)‐A‐G‐ABEA (AAG‐ABEA) to the human and rat adenosine A_1_‐receptors. Effect of VCP171 (a, c) or PD 81,723 (b, d) on the binding of BY630‐X‐(*D*)‐A‐(*D*)‐A‐G‐ABEA to the human (a, b) or rat (b, d) A_1_‐receptors. Figures show combined data from six (a) or five (b, c, d) separate experiments (each performed in triplicate). Data are expressed as mean ± SEM. *P < 0.05 fitted parameters (both *K*_D_ and *B*_max_) curves significantly different from control (without allosteric modulator; partial *F* test). ^#^ *P* < 0.05 fitted parameter for *K*_D_ significantly different from control (partial *F* test)

For the tripeptide linker variant of ABEA‐X‐BY630 (AAG‐ABEA‐X‐BY630) a similar profile was observed to that obtained with ABEA‐X‐BY630. Significant increases in specific binding were detected with VCP171 at both A_1_‐receptor species homologues (Figure 7a,c). At the human A_1_‐receptor, this appeared to be due to significant changes in both p*K*_D_ and *B*_max_ (Table 3); although for the rat homologue, this was more dependent on an increase in p*K*_D_ (Table 4). *F* test analysis of the combined data for the human A_1_‐receptor also indicated an effect of VCP171 on p*K*_D_ (Figure 7a). In the case of PD 81,723, no consistent of effect of this allosteric enhancer was observed on AAG‐ABEA‐X‐BY630 binding in the rat although a small significant increase in overall specific binding was detectable at the human A_1_‐receptor.

## 4 DISCUSSION

The data presented here have demonstrated the utility of the NanoBRET ligand binding approach to study species differences in the binding of agonists and antagonists to the adenosine A_1_‐receptor in intact living cells. The requirement of NanoBRET for close proximity between the N‐terminal Nluc tag of the receptor and the receptor‐bound fluorescent antagonist CA200645 (~10 nm) produced low levels of non‐specific binding of CA200645 in both species that was consistent with our previous work (Stoddart, Johnstone, et al., 2015). This enabled accurate determination of the binding affinities of competing A_1_‐receptor ligands to be made. This work confirmed the species differences in the affinity of DPCPX reported previously in brain membrane homogenates between the human and rat A_1_‐receptor homologues (Maemoto et al., 1997). Interestingly, the allosteric regulators PD 81,723 and VCP171 produced a small direct inhibition of CA200645 binding at high concentrations (>100 μM for PD 81,723 and 10–100 μM VCP171) that was more evident at the human A_1_‐receptor.

The most striking effects of PD 81,723 and VCP171, however, were that they significantly enhanced the binding affinities of NECA, CCPA, and adenosine to the human A_1_‐receptor, as determined from inhibition of the specific binding of CA200645 in whole cells. This was generally achieved at lower concentrations of the allosteric modulator (3–30 μM) than were required to directly inhibit the specific binding of CA200645. At the rat A_1_‐receptor, the effect of PD 81,723 on the binding affinity of adenosine and NECA was less marked and did not reach statistical significance at the concentrations tested (3–30 μM). Significant increases in the binding affinity of CCPA were, however, observed with PD 81,723 at the rat A_1_‐receptor. In contrast, VCP171 (10 and 30 μM) enhanced adenosine, CCPA, and NECA binding at the rat A_1_‐receptor. Interestingly, both modulators (3–30 μM) had no significant effect on the binding affinity of the selective A_1_‐receptor partial agonist capadenoson (Albrecht‐Kupper et al., 2012; Tendera et al., 2012), although a small effect of PD 81,723 was evident at the human receptor if the concentration of PD 81,723 was raised to 100 μM.

This apparent probe dependence is a classical feature of allosteric interactions and is consistent with VCP171 and PD 81,723 binding to a topographically distinct allosteric site on the A_1_‐receptor from which they can elicit conformational changes that lead to an alteration in the binding affinity of an agonist ligand occupying the classical orthosteric binding site (Kenakin, 2009; Keov, Sexton, & Christopoulos, 2010; Kruse et al., 2013; May, Leach, Sexton, & Christopoulos, 2007). However, taken together, the data suggest that both PD 81,723 and VCP171 can bind to both this allosteric site (leading to enhanced agonist binding) and, at higher concentrations, to the orthosteric ligand‐binding site where they are responsible for inhibiting binding of the fluorescent antagonist CA200645. Consistent with this latter observation, the recent crystal structure of the inactive human A_1_‐receptor has shown that VCP171 can be docked into the orthosteric site and its binding site overlaps with that of the classical A_1_‐receptor antagonist DPCPX (Glukhova et al., 2017). It was noticeable, however, that these authors did also identify a putative secondary binding pocket in this inactive A_1_‐receptor structure that could represent a site that is involved in allosteric regulation (Glukhova et al., 2017).

**Figure 8:**
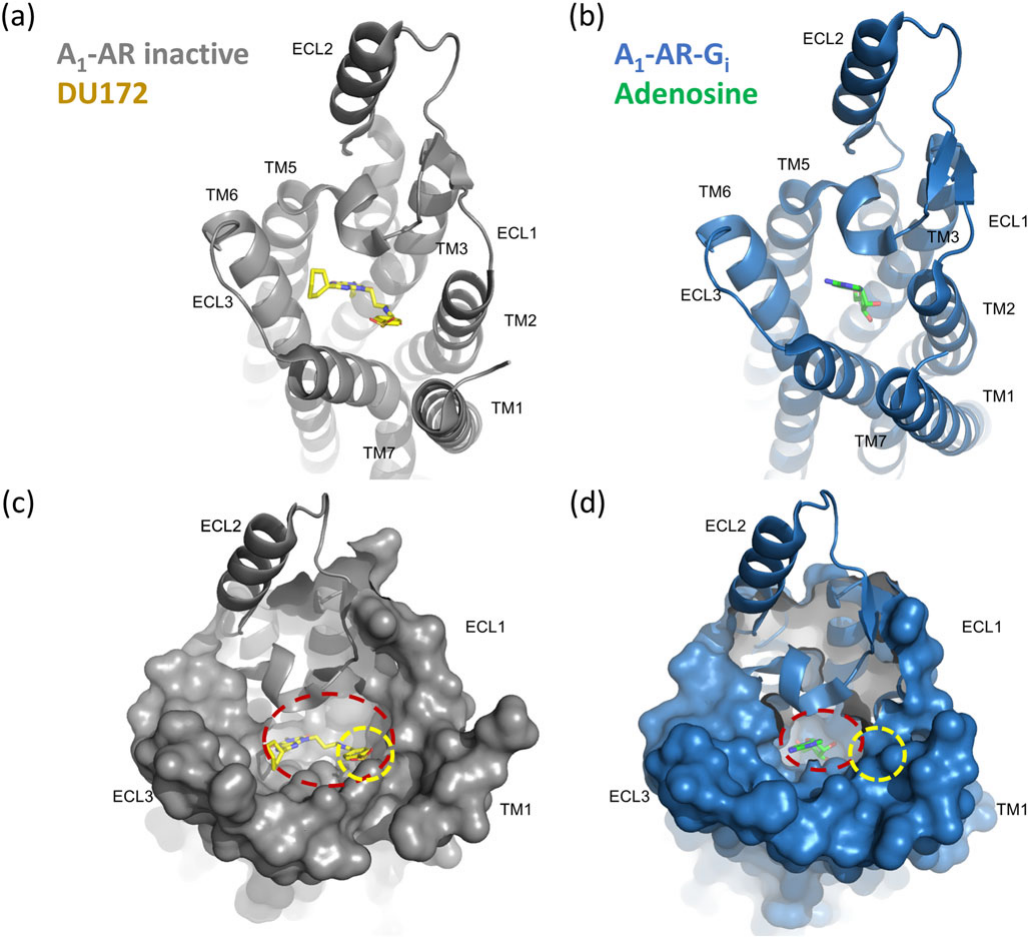
Crystal structures of the human A_1_‐receptor bound to (a, c) the orthosteric antagonist DU172 (PDB: 5UEN; Glukhova et al., 2017) or (b, d) bound to the endogenous agonist adenosine in the presence of a G_i_‐α subunit (PDB: 6D9H; Draper‐Joyce et al., 2018). Structures show the ligand binding pocket from a top‐down view. (c, d) Surface projection of the transmembrane helices, ECL1, ECL2, and ECL3 to demonstrate the closing of the binding pocket in the agonist‐bound active structure (d) compared to the antagonist‐bound inactive structure (c). The orthosteric (dashed red circle) and the position of the potential secondary allosteric binding site (dashed yellow circle) identified by Glukhova et al. (2017) in the antagonist‐bound structure are also shown (c). These positions have also been extrapolated to the agonist‐bound structure (d). 3D structures were produced using the programme PyMol (Schrodinger, Cambridge, MA, USA)

A striking feature of the inactive human A_1_‐receptor crystal structure obtained in complex with the covalently bound antagonist DU‐172 is that the ECL2 residues form an α‐helix that extends away from the transmembrane regions of the receptor in a manner that is almost perpendicular to the plane of the membrane (Glukhova et al., 2017; Figure 8a). This is a region of the receptor that mutagenesis studies have suggested is crucial to both the functional efficacy of the agonist NECA (Nguyen, Baltos, et al., 2016) and to the ability of PD 81,723 and VCP171 to elicit allosteric effects on orthosteric agonist binding (Nguyen, Vecchio, et al., 2016). It is therefore possible that ECL2 undergoes a conformational change following agonist binding to bring these residues in closer juxtaposition to the large binding pocket of the A_1_‐receptor that contains the orthosteric binding site (Glukhova et al., 2017). However, the recent structure of the adenosine‐occupied A_1_‐receptor in complex with a G_i_‐protein (Figure 8b) suggests that although the orthosteric binding cavity does collapse (and become smaller) due to an inward movement of the extracellular domains of transmembrane regions 1 and 2 (Figure 8c,d), the position of the ECL2 remains largely unaltered (Draper‐Joyce et al., 2018; Figure 8c,d). The reciprocal nature of the conformational interactions normally observed between allosteric and orthosteric sites also suggests that an orthosteric agonist needs to be present for PD 81,723 and VCP171 to bind with higher affinity to the allosteric site. Any difference in this effect between species is likely to be a consequence of subtle differences in the conformational changes induced by VCP171 and PD 81,723 in each species. This may be a consequence of their ability to induce a collapse in the orthosteric binding cavity, observed in the active crystal structure (R*), to different extents.

The lack of a significant effect of allosteric enhancers on the binding of the partial A_1_‐receptor agonist capadenoson also suggests a reduced ability of this agonist to switch the receptor from R to R* or indeed to produce a different “partially active” R* conformation. Thus, for example, recent structural information published for the β_2_‐adrenoceptor partial agonist salmeterol indicates subtle difference in the hydrogen‐bonding interactions within the orthosteric binding site for salmeterol and the full agonist adrenaline (Masureel et al., 2018). The reciprocal nature of the conformational interactions between allosteric and orthosteric sites would be consistent with a reduced influence of allosteric enhancers on the binding of capadenoson to the A_1_‐receptor.

**Figure 9:**
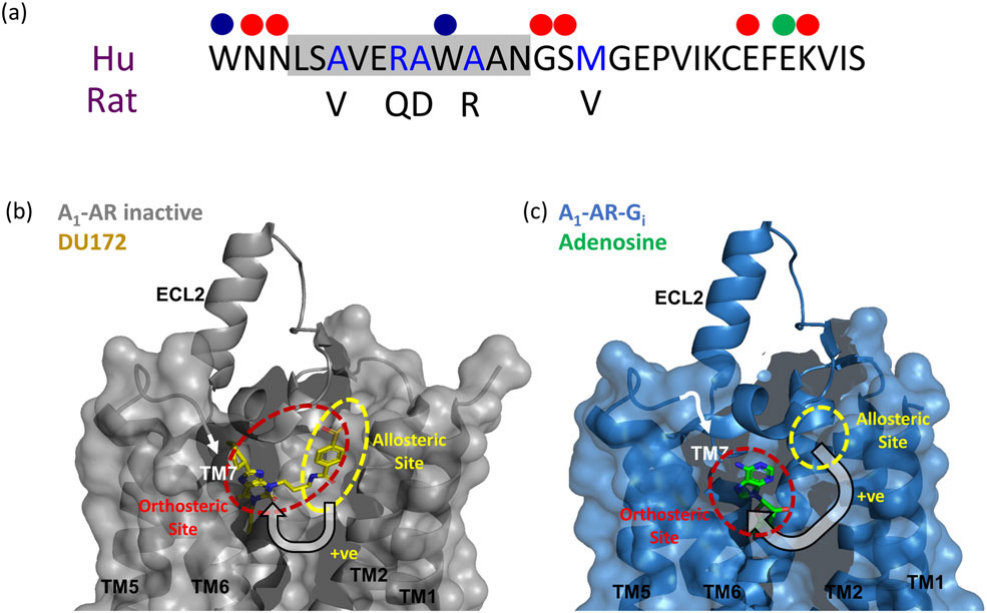
(a) Amino acid sequence of extracellular loop 2 (ECL2) of the human (Hu) A_1_‐receptor showing those residues suggested by mutagenesis studies (Nguyen, Vecchio, et al., 2016) to be involved in the allosteric effects on the binding of NECA of PD 81,723 (red circles) and VCP171 (blue circles). The helical region is highlighted in grey. E172 (green circle) is also indicated. The amino acids that differ between the human and rat ECL2 sequences are also highlighted (blue letters). (b) Crystal structure of the human A_1_‐receptor bound to the orthosteric antagonist DU172 (PDB: 5UEN; Glukhova et al., 2017) with both orthosteric and putative allosteric binding sites shown. (c) Crystal structure of the human A_1_‐receptor bound to the endogenous agonist adenosine (PDB: 6D9H; Draper‐Joyce et al., 2018) with orthosteric and allosteric binding sites shown. Note the allosteric binding site in (c) and does not overlap with the orthosteric binding site. 3D structures were produced using the program PyMol (Schrodinger, Cambridge, MA, USA)

Figure 9a shows how ECL2 differs between the two species and also highlights those residues that have been implicated in the allosteric effects of PD 81,723 or VCP171 on the binding of the agonist NECA to the human A_1_‐receptor (Nguyen, Vecchio, et al., 2016). Also highlighted is E172 that appears to be important for the direct binding of PD 81,723 and VCP171 to the human A_1_‐receptor (Nguyen, Vecchio, et al., 2016). It is notable that the mutations identified by Nguyen, Baltos, et al. (2016) do not include residues that are different between species. The amino acid sequence that represents the α‐helix of EL2 in the human sequence is highlighted in grey in Figure 9a. Helix prediction analysis using PredictProtein (Rost, Yachdav, & Liu, 2004) confirmed that there was no change in helix propensity in the two species and that the α‐helix is in the same position for the rat protein sequence. It is notable that four of the five residues that are different between rat and human A_1_‐sequence are in the α‐helical region. This suggests that the M162V change (which is adjacent to the helix domain and also to two residues mutated by Nguyen, Vecchio, et al., 2016) may underlie some of the subtle changes in allosteric action between the two species.

A direct effect of both VCP171 and PD 81,723 on agonist binding was also evident from NanoBRET studies using fluorescent A_1_‐receptor‐agonists. This was particularly clear for VCP171 where the allosteric modulator significantly increased the level of specific binding of all three fluorescent agonists tested at the human A_1_‐receptor. This was a consequence of both an increase in maximal binding capacity (*B*_max_) and affinity (p*K*_D_). Consistent with this positive allosteric effect, we have previously shown that PD 81,723 (10 μM) can slow the dissociation of BY630‐ABA from human A_1_‐receptors expressed in CHO cells (May et al., 2010). The effect of VCP171 was, however, also species dependent with an increase in both binding parameters evident with all three fluorescent agonists at the human A_1_‐receptor, but the effects were limited to *B*_max_ (ABEA‐X‐BY630) and p*K*_D_ (AAG‐ABEA‐X‐BY650) for particular fluorescent agonists at the rat species homologue. It is also worth pointing out that our results suggest that the functionalization and addition of fluorophore to the agonist chemical scaffold has been achieved at a point that does not clash with the allosteric modulator binding site.

The data obtained with VCP171 at the human A_1_‐receptor are consistent with an increase in the proportion of a higher affinity agonist conformation (R*; Figure 9c) that was only detectable with fluorescent A_1_‐receptor agonists. That is, these fluorescent analogues of adenosine and NECA have a lower affinity for the antagonist‐bound conformation of the receptor (R; Figure 9b) and do not detect binding to this conformation at the concentrations used in the present fluorescent agonist‐binding studies. The difference in the extent to which VCP171 and PD 81,723 enhance fluorescent agonist‐binding *B*_max_ values might also suggest that this property underlies the ability of allosteric enhancers to produce direct agonist actions in the absence of orthosteric agonists (Nguyen, Vecchio, et al., 2016). Thus, the increased formation of active receptor conformations (R*) in the presence of the allosteric regulator may lead to increased stimulation of intracellular signalling pathways. An increased conversion of inactive receptor (R) to active receptor conformations (R*) by VCP171 and PD 81,723 may also explain the decrease in specific binding of the fluorescent antagonist CA200645 observed above, although the structure of the inactive receptor also indicates that the allosteric ligands can bind to the orthosteric binding site (Glukhova et al., 2017).

In summary, the present study has shown that PD 81,723 and VCP171 can elicit positive allosteric effects on the binding affinity of orthosteric agonists at both the rat and human adenosine A_1_‐receptors. This work also confirms that these two allosteric regulators exhibit both probe and species homologue dependence. Thus, the allosteric effect on the highly selective partial agonist capadenoson is much less marked than for the full agonists NECA and adenosine in both species. In addition, at higher concentrations, both allosteric regulators have a direct inhibitory effect on the binding of the orthosteric fluorescent antagonist CA200645 that is consistent with the suggestion from crystallographic studies that indicates that they can also bind directly to the orthosteric binding site of the A_1_‐receptor. Finally, VCP171 and, to a lesser extent, PD 81,723, were also able to increase the specific binding of three fluorescent A_1_‐receptor agonists in a species‐dependent manner that involved increases in *B*_max_ and p*K*_D_. This latter effect may provide new insights into the mechanisms by which allosteric enhancers can elicit functional responses in the absence of orthosteric A_1_‐receptor agonists.

## CONFLICT OF INTEREST

The authors declare no conflicts of interest.

## AUTHOR CONTRIBUTIONS

S.L.C., M.S., J.W., and S.J.H. participated in the research design. M.J. and P.J.S. synthesized VCP171 and VCP746. S.L.C. conducted the experiments. S.L.C., M.S., and S.J.H. performed the data analysis. S.L.C., M.S., P.J.S., J.W., and S.J.H. wrote or contributed to the writing of the manuscript.

## DECLARATION OF TRANSPARENCY AND SCIENTIFIC RIGOUR

This Declaration acknowledges that this paper adheres to the principles for transparent reporting and scientific rigour of preclinical research as stated in the *BJP* guidelines for Design & Analysis, and as recommended by funding agencies, publishers and other organisations engaged with supporting research.
